# Diversity, prevalence, and expression of cyanase genes (*cynS*) in planktonic marine microorganisms

**DOI:** 10.1038/s41396-021-01081-y

**Published:** 2021-08-18

**Authors:** Xuewei Mao, Jianwei Chen, Cock van Oosterhout, Huan Zhang, Guangxing Liu, Yunyun Zhuang, Thomas Mock

**Affiliations:** 1 Key Laboratory of Environment and Ecology, Ministry of Education, Ocean University of China, Qingdao, 266100 China; 2 Laboratory for Marine Ecology and Environmental Science, Qingdao National Laboratory for Marine Science and Technology, Qingdao, 266237 China; 3 School of Environmental Sciences, University of East Anglia, Norwich Research Park, NR4 7TJ Norwich, UK; 4 BGI-Qingdao, BGI-Shenzhen, Qingdao, 266555 China; 5 Qingdao-Europe Advanced Institute for Life Sciences, BGI-Shenzhen, Qingdao, 266555 China; 6 Department of Marine Sciences, University of Connecticut, Groton, CT 06340 USA

**Keywords:** Microbial biooceanography, Water microbiology

## Abstract

Cyanate is utilized by many microbes as an organic nitrogen source. The key enzyme for cyanate metabolism is cyanase, converting cyanate to ammonium and carbon dioxide. Although the cyanase gene *cynS* has been identified in many species, the diversity, prevalence, and expression of *cynS* in marine microbial communities remains poorly understood. Here, based on the full-length cDNA sequence of a dinoflagellate *cynS* and 260 homologs across the tree of life, we extend the conserved nature of cyanases by the identification of additional ultra-conserved residues as part of the modeled holoenzyme structure. Our phylogenetic analysis showed that horizontal gene transfer of *cynS* appears to be more prominent than previously reported for bacteria, archaea, chlorophytes, and metazoans. Quantitative analyses of marine planktonic metagenomes revealed that *cynS* is as prevalent as *ureC* (urease subunit alpha), suggesting that cyanate plays an important role in nitrogen metabolism of marine microbes. Highly abundant *cynS* transcripts from phytoplankton and nitrite-oxidizing bacteria identified in global ocean metatranscriptomes indicate that cyanases potentially occupy a key position in the marine nitrogen cycle by facilitating photosynthetic assimilation of organic N and its remineralisation to NO_3_ by the activity of nitrifying bacteria.

Cyanate (OCN^−^) is an oxidation product of cyanide and a decomposition product of urea [1, 2]. It is considered as an organic nitrogen source for diverse prokaryotic and eukaryotic microbes in terrestrial and aquatic ecosystems [2–7] with concentrations in the nanomolar range [4, 7–9]. Cyanate is also formed intracellularly from urea and carbamoyl phosphate, making it part of the central nitrogen metabolism [10–12]. In spite of the central metabolic role of cyanate, it has received much less attention than other organic nitrogen compounds, particularly for marine environments. However, it has been found that cyanate is likely an essential N source for cyanobacteria in oligotrophic oceans [2, 6, 13, 14] and an alternate N substrate for marine nitrification and anammox [15–17].

Cyanate metabolism relies on the well-characterized enzyme cyanase, which catalyzes the reaction of cyanate with bicarbonate to produce ammonium and carbon dioxide [18]. The cyanase gene *cynS* has been identified in many terrestrial and aquatic species and was reported to play a significant role in the assimilation of exogenous cyanate and detoxification of internally generated cyanate [6, 13, 16, 19–28]. However, knowledge on the diversity and evolution of *cynS* in marine microbes is rather limited, including its prevalence in the oceanic system.

To address this knowledge gap, we retrieved 260 *cynS* homologs across the tree of life (Table S1) based on the full-length cDNA from the marine dinoflagellate *Alexandrium pacificum* (*APcynS*, 653 bp, GenBank accession number: MZ666876) (Fig. S1; Table S2) and its deduced amino acid sequence (APcyanase). This reference dataset was used to query marine metagenomes and metatranscriptomes using the Ocean Gene Atlas (OGA) [29] to explore the biogeography and in situ expression pattern of *cynS* (full methods were described in supplements).

An amino-acid alignment composed of 260 homologs revealed the presence of nine ultra-conserved residues (Fig. 1a), potentially responsible for the catalytic activity and structural stability. Six of them have not been documented before [25, 30–32]. Modeling of the 3D enzyme structure indicated the following subunit organization: a decameric holoenzyme with a core formed by five dimers [30, 31] (Fig. 1b, f; Fig. S2). Five active sites are located between dimers forming an inner ring (Fig. 1c, g), non-covalently bound with five oxalate di-anions (Fig. 1d, e, h, i; Table S3), indicating the possible binding sites of cyanate. In *E. coli*, there are four types of residue-oxalate interactions known for binding cyanate (Fig. 1j) [30]. However, only type 1 is present in modeled APcyanase (Fig. 1j), suggesting reduced plasticity in binding cyanate. Whether this structural variation translates into different binding affinities and therefore potentially physiological roles of the APcyanase remains to be seen.

**Fig. 1:**
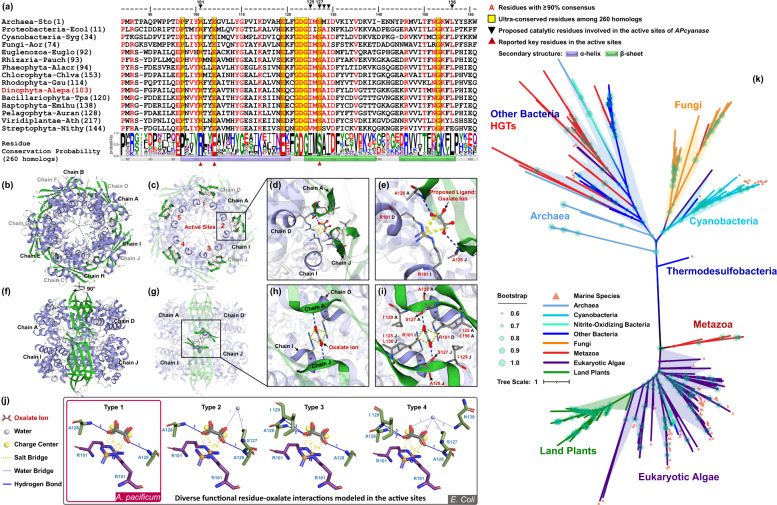
Alignment, quaternary structure, proposed catalytic residues, and phylogenetic analysis of cyanases. **a** Alignment of the catalytic domain in cyanases from representative species. Numbers in parentheses refer to the sequence ID from the full list (Table S1). **b** The front view of the decameric cyanase from the dinoflagellate *Alexandrium pacificum*. Alpha-helix and beta-hairpin is shown in purple and green, respectively. Ten monomers are labeled as chains A-J. **c** Overall location of the five active sites. **d**, **e** Enlarged views of the chain interactions and residues-oxalate anion interactions. Ball-and-stick colored in red and gray denotes oxalate anions. **f** Structures flipped 90° clockwise around the Y-axis shows the view of two dimer pairs. **g** Side view of one active site. **h**, **i** Enlarged views of proposed catalytic residues. **j** Four types of residue-oxalate anion interactions in cyanases. **k** Unrooted maximum likelihood tree of cyanases. Only bootstrap values ≥60% were shown. Lineages are color-coded and marine species are labeled with triangles.

Four major clades of cynases were identified based on their phylogenic relationships (Fig. 1k, Fig. S3). Interestingly, horizontal gene transfer (HGT) of *cynS* contributed to the evolution of bacteria, archaea, and eukaryotes including microalgae (*Bathycoccus*, *Micromonas*) and metazoans, which provides evidence that HGT of *cynS* is more common than previously documented [3, 6, 13, 16, 27, 33–35].

To contextualize cyanate metabolism in the upper ocean from the surface (epipelagic) down to the intermediate depths of ca. 1000 m (mesopelagic (MES)), we analyzed the prevalence and expression of *cynS* in comparison to *ureC*, the gene encoding the urease subunit alpha. The urea cycle, unlike cyanate metabolism, is well studied in many marine microbes including the acquisition of urea as an organic nitrogen source. Homologs of both genes and their corresponding transcripts could be retrieved from almost all sampling stations of the OGA (Table S4), which suggests their overall prevalence in many marine microbes. However, the normalized gene activity of *cynS* and *ureC* differed depending on the size class, taxonomic group and the water depth (Fig. 2; Fig. S4). Interestingly, the transcriptional activity of *ureC* appears to be much lower compared to *cynS* in the larger size fraction (0.8–2000 μm) mostly representing eukaryotic microbes and for both, surface (SRF) and deep chlorophyll maximum (DCM) (Fig. 2a, b, f, g). Pelagophytes, dinophytes, bacillariophytes, and fungi contributed the most *cynS* transcripts in the epipelagic ocean with pelagophyte transcripts dominating the surface layer (Fig. 2a, b). Transcripts for both genes in the smaller size fraction (0.22–3 μm) were mostly derived from prokaryotes (Figs. 2c–e, 2h-j). In the surface ocean, *Synechococcus* contributed most of the *cynS* transcripts in non-polar oceans whereas picochlorophyte *cynS* transcripts were most dominant in the coastal Arctic (Fig. 2c). In contrast, proteobacteria together with unclassified microbes contributed most of the *ureC* transcripts in surface ocean metatranscriptomes regardless of geography (Fig. 2h). The taxonomic contributions of the *ureC* transcripts did not change much for the DCM and not even the MES zone although Gammaproteobacteria appear to have contributed more *ureC* transcripts in the MES compared to the epipelagic (Fig. 2i, j). By comparison, unclassified microbes together with *Prochlorococcus* contributed more *cynS* transcripts in the DCM (Fig. 2d). For the MES, most of the *cyn*S transcripts were contributed by unclassified microbes, Nitrospinae and Proteobacteria (Fig. 2e).

**Fig. 2:**
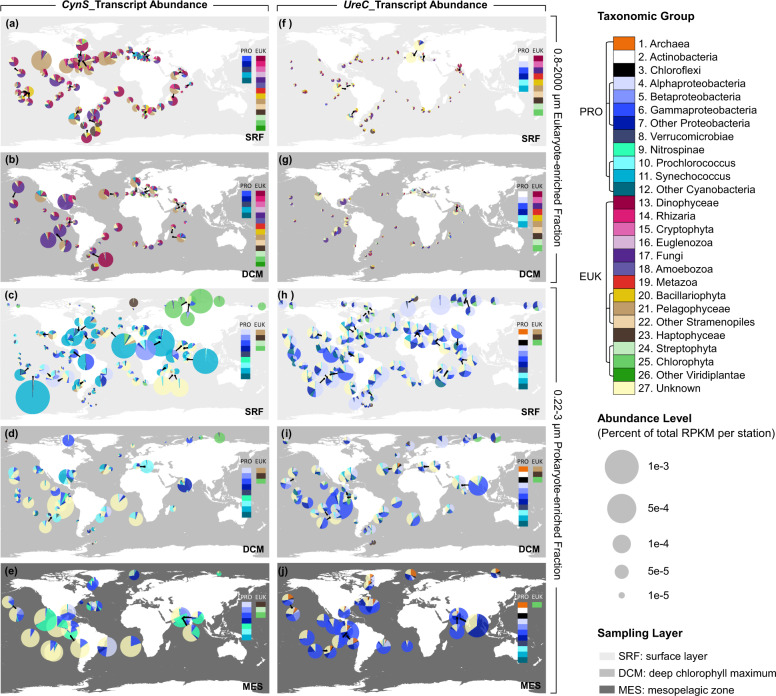
Biogeographic distribution and taxonomic composition of *cynS* and *ureC* transcripts in the global ocean from the surface (SRF) to the deep chlorophyll maximum (DCM) and the mesopelagic (MES). *CynS* (**a**–**e**) and *ureC* (**f**–**j**) in eukaryote-enriched (0.8–2000 μm fraction) and prokaryote-enriched (0.22–3 μm fraction) metatranscriptomes. Samples from different size fractions have been pooled in each station. No data are available for 0.8–2000 μm fraction in MES.

The abundance of *cynS* transcripts from most prokaryotic and eukaryotic phytoplankton was negatively correlated (*p* < 0.05) with dissolved inorganic nitrogen concentrations (Fig. S5a, b; Table S5a, b). The latitudinal differences in the taxonomic contributions of *cynS* correlated negatively with temperature (*p* < 0.05), suggesting that cyanases are induced in picochlorophytes by low temperature. As no Arctic samples were available for the larger size fraction (0.8–2000 μm) representing mainly eukaryotes, it remains elusive if this group of organisms does have similar *cynS* expression patterns under polar conditions.

Although much less *cynS* transcripts were detected in the MES, the contribution of Nitrospinae was more significant compared to the epipelagic (Fig. 2e). Members of the phylum Nitrospinae are known to be the most abundant nitrite-oxidizing bacteria (NOB) in the oceans with an important role in dark-ocean carbon fixation [3, 16]. Cyanate metabolism of NOBs is common and essential for the global nitrogen cycle, supplying ammonia oxidizers with ammonium, which is nitrified by this nitrifying consortium including NOBs [3]. In our study, *cynS* transcripts from Nitrospinae in the epipelagic layers were limited to only few stations in the Eastern Pacific and Arctic Ocean (Fig. 2c, d). However, more prevalent and abundant were these transcripts in the MES (Fig. 2e). The abundance of *cynS* transcripts from Nitrospinae was positively correlated with nitrate and nitrite (*p* < 0.05, Fig. S5b; Table S5b), suggesting that cyanate metabolism in Nitrospinae may facilitate marine nitrification. In contrast, *cynS* was not detected in marine ammonia-oxidizing archaea of the phylum Thaumarchaeota. However, the *ureC* transcript from this taxon was detected mainly in MES zone (Fig. 2j) and positively correlated with depth and the concentration of nitrate (*p* < 0.05, Fig. S5b, Table S6b). This corroborates previous findings as marine Thaumarchaeota genomes lack the canonical *cynS* gene but the organisms can utilize cyanate and urea to fuel nitrification [15]. The contents of all the retrieved unigenes from OGA have been summarized in Supplementary Tables S7–S14.

Taken together, *cynS* is a conserved gene ubiquitous across the tree of life, transferred frequently via HGT. Comparative analyses based on the prevalence and expression of *cynS* and *ureC* representing intertwined processes of organic N metabolism in marine microbes suggest that cyanate is at least as important as urea in the oceans. Cyanate likely supports the assimilation of organic N in photoautotrophs when inorganic N is scarce and it appears to contribute to remineralisation by the activity of nitrifying bacteria which produce nitrate in deeper layers of the oceans.

## Supplementary information

Supplementary Material and Methods

Supplementary figure legends

Supplementary table S1

Supplementary table S2

Supplementary table S3

Supplementary table S4

Supplementary table S5

Supplementary table S6

Supplementary table S7-S14

Supplementary figure 1

Supplementary figure 2

Supplementary figure 3

Supplementary figure 4

Supplementary figure 5
