# Supplementary material for: Diversity, prevalence, and expression of cyanase genes (cynS) in planktonic marine microorganisms
Source: ISME J. 2021 Aug 18;16(2):602–5. doi: 10.1038/s41396-021-01081-y (PMC8776842; doi:10.1038/s41396-021-01081-y)
Supplement: Supplementary file 1 — Supplementary Material and Methods [file 41396_2021_1081_MOESM1_ESM.docx]

# **Diversity, prevalence, and expression of cyanase genes (*cynS*) in planktonic marine microorganisms**

# **Supplementary Materials and Methods**

**RNA isolation, cDNA synthesis, *APcynS* cloning and sequencing**

Laboratory cultured cells (~10^6^) of the *Alexandrium pacificum* (AP) strain ACHK were harvested by centrifugation at 1500g at 20℃ for 10 minutes. Total RNA was extracted using the Trizol method^[1]^ and purified with Quick-RNA MiniPrep Kit (Zymo Research, USA). The RNA was quantified with a NanoDrop 2000c (ThermoFisher Scientific, USA). First-strand cDNA was synthesized with the Superscript III reverse transcriptase and modified oligo-dT^[2]^ from purified RNA. The DinoSL-T7N full-length double-strand cDNA was subsequently synthesized^[2]^. The quality and integrity of the cDNA was checked on 1% w/v agarose gel. *CynS*-specific primers (Supplementary Table S2) were designed based on the *Alexandirum* transcriptome^[3]^ to obtain the full-length cDNA of the *APcynS*. PCR was performed using DinoSL, *cynS*-specific primers, and GeneRacer3. PCR products were purified and cloned into a pCR^™^4-TOPO^™^ TA vector (Invitrogen, ThermoFisher Scientific, USA) for sequencing.

**APcyanase structure analyses**

The amino acid sequence of the APcyanase was deduced by using the EMBOSS Transeq Translation Server (EMBL-EBI)^[4]^ and annotated by InterPro^[5]^. The secondary structure was predicted using Jpred 4 and Phyre2^[6, 7]^. The tertiary and quaternary structure modelling was performed using SWISS-MODEL^[8, 9, 10]^ including a template search, model building, model quality estimation, ligand modelling and assessing the oligomeric state conservation. *Escherichia coli* (strain K12) cyanase (PDB code: 1dwk) was used as the modelling template. APcyanase interactions in active sites were predicted by SWISS-MODEL and annotated with PLIP^[11]^. Protein-ligand interactions were summarized in Supplementary Table S3. The APcyanase model and its structure details were analyzed by PyMOL v2.3.3^[12]^.

**Recruitment of the 260 *cynS* homologs**

We first retrieved cyanase and *cynS* sequences from representative species of archaea, bacteria, fungi and plants, which have been annotated according to EC:4.2.1.104 available in KEGG and Pfam. For eukaryotic algae, *cynS* sequences were retrieved from the algal genomes annotated in JGI’s PhycoCosm and the KEGG database. To thoroughly retrieve and investigate the eukaryotic algal *cynS*, the translated APcyanase from full-length cDNA sequence of *APcynS* was used as a query to tBLASTn (e-value≤1e-5) against NCBI GenBank’s (nr/nt) collection, the transcriptome shotgun assembly (TSA), China National Gene Bank (CNGB) and *Porphyra* ESTs databases. The annotation and functional domains of all retrieved sequences were carried out through the UniProt database. Only sequences with a nearly complete coding sequence (CDS), and with exact cyanase domain structure including conserved motifs were selected for subsequent analyses, yielding a total of 260 homologs (Supplementary Table S1). The amino acid sequences were used for phylogenetic analysis.

**Sequence alignment and comparison**

Amino acid sequences of 260 homologs were used to identify ultra-conserved sites and the diversity of *cynS*. Multiple amino acid sequence alignments were performed using MEGA6^[13]^ and MultAlin^[14]^. Conserved amino acids were highlighted by WebLogo-v3^[15]^.

**Phylogenetic analyses**

A total of 260 single-copy amino acid sequences were aligned using MEGA6^[13]^ and ClustalX v2^[16]^. Maximum likelihood was used to generate the tree in PhyML-v3^[17]^ under the best model: LG+G+I. All bootstrap values were determined using 100 repeats of the initial alignment. Only bootstrap values ≥60% were shown. The resulting tree was labeled using MEGA6^[13]^and iTOL v4^[18]^.

**Ocean Gene Atlas**

***CynS* and *ureC* homologs detection and annotation**

To retrieve homologs of *cynS* from the Ocean Gene Atlas, a hidden Markov model (HMM) profile was generated as the query from the Pfam cyanase database (Pfam ID 02560) including the set of 260 homologs using the HMMER v3.3 package^[19]^. For *ureC*, Pfam HMM (Pfam ID 00449) was downloaded directly from the Pfam website as the query. Hmmer method was selected for cyanase protein homology searches under default settings with the expect threshold of 1E-3. The eukaryote-enriched Marine Atlas of the Tara Ocean Unigenes (MATOU)^[22]^ and the prokaryote-enriched Oceans Microbiome Reference Gene Catalog (OM-RGC.v2)^[23]^ were queried in Ocean Gene Atlas^[24]^. The output included alignment results, homolog sequences FASTA files, normalized abundance of the homologs, and environmental data. An InterProScan search^[25]^ was performed to re-annotate the retrieved homologs. Only hits annotated as cyanase (PF02560) and urease (PF01979, amidohydro_1; PF00449, urease_alpha; PF00699, urease_beta; PF00547, urease_gamma) with the e-value <1e-5 were used for subsequent analyses. The gene list, alignment results, taxonomic affiliation and fasta sequences are provided in Supplementary Tables S7-S14. Their biogeographic distribution was plotted in R (v. 3.4.1) using scatterpie and ggplot2^[20]^.

**Computation of unigene abundance/expression and taxonomic community composition**

Unigene abundance/expression was computed as RPKM (reads per kilo base covered per million of mapped reads) and two normalization procedures were performed depending on the analyses. For the biogeographic distribution pie chart, ‘percent of total genetic RPKM per sample’ normalization was applied, i.e., dividing the sum of the abundance of *cynS* homologs by the sum of the total gene abundance for the same sample. Samples from different size fractions have been pooled for calculating the total abundance level in each station. For taxon-specific gene expression/abundance in correlation analyses, RPKM of each unigene was divided by that of all genes from the same taxon.

**Spearman’s correlation analyses**

Correlation analysis between each taxonomic group’s gene expression/abundance and various environmental variables were conducted by computing Spearman's correlation coefficient (Rho, ρ) in the R (v. 3.4.1) package corTest^[20]^. The total unigenes’ expression/abundance value per sample were merged before conducting correlation analyses. Results were showed in heatmaps^[21]^ using the following threshold criteria: n > 20, cluster method=complete, distance methods=euclidean, branch form=cladogram. Significance levels (p < 0.05) were indicated in the heatmaps. Overall correlation results can be found in Supplementary Tables S5-S6. Results were plotted using TBtools (v. 1.082)^[21]^.

**Data availability**

The full-length cDNA sequence of *cynS* from *A. pacificum* is shown in Fig. S1 and will be submitted to NCBI upon the acceptance of this paper. Information about the 260 *cynS* reference catalog is available in Supplementary Table S1. Information about *cynS* and *ureC* unigenes retrieved from Ocean Gene Atlas ([https://tara-oceans.mio.osupytheas.fr/ocean-gene-atlas/)^[24^](https://tara-oceans.mio.osupytheas.fr/ocean-gene-atlas/)%5b24)^]^ is provided in Supplementary Tables 7-14. The original gene catalog, distribution of the sampling stations and methodologies are accessible at: <http://www.genoscope.cns.fr/tara/> (MATOU) and <https://www.ocean-microbiome.org/> (OM-RGC.v2). Environmental parameters are available from PANGAEA: <https://doi.org/10.1594/PANGAEA.875582>.

**Supplementary References**

1. Zhang H, Zhuang Y, Gill J, Lin S. Proof that dinoflagellate spliced leader (DinoSL) is a useful hook for fishing dinoflagellate transcripts from mixed microbial samples: Symbiodinium kawagutii as a case study. Protist. 2013;164: 510-527.

2. Zhang H, Hou Y, Miranda L, Campbell DA, Sturm N R, Gaasterland T, et al. Spliced leader RNA trans-splicing in dinoflagellates. P Natl Acad Sci USA. 2007;104: 4618-4623.

3. Zhuang Y, Zhang H, Hannick L, Lin S. Metatranscriptome profiling reveals versatile N-nutrient utilization, CO2 limitation, oxidative stress, and active toxin production in an Alexandrium fundyense bloom. Harmful Algae. 2015;42: 60-70.

4. Madeira F, Park YM, Lee J, Buso N, Gur T, Madhusoodanan N, et al. The EMBL-EBI search and sequence analysis tools APIs in 2019. Nucleic Acids Res. 2019;47: W636-W641.

5. Blum M, Chang HY, Chuguransky S, Grego T, Kandasaamy S, Mitchell A, et al. The InterPro protein families and domains database: 20 years on. Nucleic Acids Res. 2021;49: D344-D354.

6. Drozdetskiy A, Cole C, Procter J, Barton GJ. JPred4: a protein secondary structure prediction server. Nucleic Acids Res. 2015;43: W389-W394.

7. Kelley LA, Mezulis S, Yates CM, Wass MN, Sternberg MJ. The Phyre2 web portal for protein modeling, prediction and analysis. Nat Protoc. 2015;10: 845-858.

8. Waterhouse A, Bertoni M, Bienert S, Studer G, Tauriello G, Gumienny R, et al. SWISS-MODEL: homology modelling of protein structures and complexes. Nucleic Acids Res. 2018;46: W296-W303.

9. Guex N, Peitsch MC, Schwede T. Automated comparative protein structure modeling with SWISS‐MODEL and Swiss‐PdbViewer: A historical perspective. Electrophoresis. 2009;30: S162-S173.

10. Bienert S, Waterhouse A, de Beer TA, Tauriello G, Studer G, Bordoli, L, et al. The SWISS-MODEL Repository—new features and functionality. Nucleic Acids Res. 2017;45: D313-D319.

11. Salentin S, Schreiber S, Haupt VJ, Adasme MF, Schroeder M. PLIP: fully automated protein-ligand interaction profiler. Nucleic Acids Res. 2015;43: W443-W447.

12. DeLano WL. The PyMOL molecular graphics system. http://www.pymol.org. 2002.

13. Tamura K, Stecher G, Peterson D, Filipski A, Kumar S. MEGA6: molecular evolutionary genetics analysis version 6.0. Mol Biol Evol. 2013;30: 2725-2729.

14. Corpet F. Multiple sequence alignment with hierarchical clustering. Nucleic Acids Res. 1988;16: 10881-10890.

15. Crooks GE, Hon G, Chandonia JM, Brenner SE. WebLogo: a sequence logo generator. Genome Res. 2004;14: 1188-1190

16. Larkin MA, Blackshields G, Brown NP, Chenna R, McGettigan PA, McWilliam H, et al. Clustal W and Clustal X version 2.0. Bioinformatics. 2007;23: 2947-2948.

17. Guindon S, Dufayard JF, Lefort V, Anisimova M, Hordijk W, Gascuel O. New algorithms and methods to estimate maximum-likelihood phylogenies: assessing the performance of PhyML 3.0. Syst Biol. 2010;59: 307-321.

18. Letunic I, Bork P. Interactive Tree Of Life (iTOL) v4: recent updates and new developments. Nucleic Acids Res. 2019;47: W256-W259.

19. Wheeler TJ, Eddy SR. nhmmer: DNA homology search with profile HMMs. Bioinformatics. 2013;29: 2487-2489.

20. Team RC. R: A language and environment for statistical computing. 2013.

21. Chen C, Chen H, Zhang Y, Thomas HR, Frank MH, He Y, et al. TBtools: an integrative toolkit developed for interactive analyses of big biological data. Mol Plant. 2020;13: 1194-1202.

22. Carradec Q, Pelletier E, Da Silva C, Alberti A, Seeleuthner Y, Blanc-Mathieu R, et al. A global ocean atlas of eukaryotic genes. Nat Commun. 2018;9: 1-13.

23. Salazar G, Paoli L, Alberti A, Huerta-Cepas J, Ruscheweyh HJ, Cuenca M, et al. Gene expression changes and community turnover differentially shape the global ocean metatranscriptome. Cell. 2019;179: 1068-1083.

24. Villar E, Vannier T, Vernette C, Lescot M, Cuenca M, Alexandre A, et al. The Ocean Gene Atlas: exploring the biogeography of plankton genes online. Nucleic Acids Res. 2018;46: W289-W295.

25. Zdobnov EM, Apweiler R. InterProScan–an integration platform for the signature-recognition methods in InterPro. Bioinformatics. 2001;17: 847-848.
