# Supplementary material for: Diversity, prevalence, and expression of cyanase genes (cynS) in planktonic marine microorganisms
Source: ISME J. 2021 Aug 18;16(2):602–5. doi: 10.1038/s41396-021-01081-y (PMC8776842; doi:10.1038/s41396-021-01081-y)
Supplement: Supplementary file 2 — Supplementary figure legends [file 41396_2021_1081_MOESM2_ESM.docx]

**Fig. S1** *APcynS* full-length cDNA sequence and deduced amino acid sequence. 5’-UTR and 3’-UTR are shaded in grey. Dinoflagellate spliced leader (DinoSL) is marked in purple. Start codon and stop codon are highlighted in yellow.

**Fig. S2** Homology modelling alignment of APcyanase (gold) and ECcyanase (*E. coli*, silver). The monomer and decamer of APcyanase were predicted using SWISS-MODEL. The figure was generated with PyMOL. ‘N’ and ‘C’ in monomer represent N-terminal and C-terminal respectively. ECcyanase (PDB: 1dwk) was used as the structural template. Red-and-green sticks represent the oxalate ion residues from both APcyanase and ECcyanase. Red-and-yellow balls represent the non-functional sulfate ion from ECcyanase. Pink dots indicate non-functional chloride ions from ECcyanase. Color-coded sequences indicate the modelled secondary structure.

**Fig. S3** Unrooted maximum-likelihood tree of cyanase homologs. Branches of different lineages are color coded and labelled with each species taxonomy ID summarized in Table S1. Taxa names in colors indicate horizontal gene transfer events. Branch lengths were estimated by maximum likelihood under a model with Akaike information criterion. Size of the circles at the nodes represents bootstrap values and maximum likelihood support. Only bootstrap values ≥60% are shown. Marine species are labelled with red triangles.

**Fig. S4** Genetic abundance, the biogeographic distribution and taxonomic composition of *cynS* and *ureC* in the global ocean from the surface (SRF) to the deep chlorophyll maximum (DCM) and the mesopelagic (MES). *CynS*(a-e) and *ureC* (f-j) gene abundance and taxonomic composition are from eukaryote-enriched (0.8-2000μm fraction, MATOU) and prokaryote-enriched (0.22-3μm fraction, OMRGC.v2) metagenomic datasets. Samples from different size fractions have been pooled in each station. No data for MES 0.8-2000μm fraction.

**Fig. S5** Heatmaps of correlations between environmental parameters and gene expression level/abundance among key taxonomic groups from eukaryote-enriched (a-b) and prokaryote-enriched sampling (c-d). Spearman’s correlation coefficients (ρ, Rho) are used for comparison, significant levels (*p* ≤ 0.05) are labelled with circles. Correlation data can be found in Supplementary Tables S5-S6. The detailed description of the methodology and environmental data are available at: <https://www.ocean-microbiome.org/> and <https://doi.org/10.1594/PANGAEA.875582>.

**Supplementary Table S1** Catalog of the 260 cyanase homologs.

**Supplementary Table S2** Primers used in the full-length *APcynS* cDNA synthesis and nested PCR.

**Supplementary Table S3** Key features of the reported protein-ligand interactions in cyanases.

**Supplementary Table S4** Statistics of sampling station and sample number in different water layers.

**Supplementary Table S5** Spearman's correlation between environmental parameters and *cynS* gene expression level (metaT) or gene abundance (metaG). Spearman's correlation coefficients were denoted under given color scheme. Significant *p*-value (*p*<0.05) were labelled in blue. n=the number of the scatters.

**Supplementary Table S6** Spearman's correlations between environmental parameters and *ureC* gene expression level (metaT) or gene abundance (metaG). Spearman's correlation coefficients were denoted under given color scheme. Significant *p*-value (*p*<0.05) were labelled in blue. n=the number of the scatters.

**Supplementary Table S7** *CynS* gene list from the Ocean Gene Atlas (OM-RGC_v2_metaT), the taxonomic assignment, identification e-value and sequences used in this study.

**Supplementary Table S8** *CynS* gene list from the Ocean Gene Atlas (OM-RGC_v2_metaG), the taxonomic assignment, identification e-value and sequences used in this study.

**Supplementary Table S9** *CynS* gene list from the Ocean Gene Atlas (MATOU_v1_metaT), the taxonomic assignment, identification e-value and sequences used in this study.

**Supplementary Table S10** *CynS* gene list from the Ocean Gene Atlas (MATOU_v1_metaG), the taxonomic assignment, identification e-value and sequences used in this study.

**Supplementary Table S11** *UreC* gene list from the Ocean Gene Atlas (OM-RGC_v2_metaT), the taxonomic assignment, identification e-value and sequences used in this study.

**Supplementary Table S12** *UreC* gene list from the Ocean Gene Atlas (OM-RGC_v2_metaG), the taxonomic assignment, identification e-value and sequences used in this study.

**Supplementary Table S13** *UreC* gene list from the Ocean Gene Atlas (MATOU_v1_metaT), the taxonomic assignment, identification e-value and sequences used in this study.

**Supplementary Table S14** *UreC* gene list from the Ocean Gene Atlas (MATOU_v1_metaG), the taxonomic assignment, identification e-value and sequences used in this study.
