# Supplementary material for: Diversity, prevalence, and expression of cyanase genes (cynS) in planktonic marine microorganisms
Source: ISME J. 2021 Aug 18;16(2):602–5. doi: 10.1038/s41396-021-01081-y (PMC8776842; doi:10.1038/s41396-021-01081-y)
Supplement: Supplementary file 10 — Supplementary figure 1 [file 41396_2021_1081_MOESM10_ESM.pdf]

```
     DinoSL                                                        M  A  E
  1 TCCGTAGCCATTTTGGCTCAAGCAGCGGAAGCTTCCAGAGAGGGGCAGGAAGATGGCGGA 60
    ----:----|----:----|----:----|----:----|----:----|----:----|
     L  P  P  A  K  R  A  R  V  D  D  E  K  A  T  L  V  A  R  L
 61 GCTACCGCCGGCCAAGCGGGCTCGGGTGGACGATGAGAAGGCCACCCTGGTGGCGCGGCT 120
    ----:----|----:----|----:----|----:----|----:----|----:----|
     L  A  A  K  E  A  S  G  K  S  F  D  E  I  A  A  A  L  G  L
121 CCTGGCAGCCAAGGAGGCCAGCGGCAAGTCCTTCGACGAGATCGCGGCGGCCCTGGGCCT 180
    ----:----|----:----|----:----|----:----|----:----|----:----|
     T  N  A  Y  T  A  N  L  F  F  N  Q  A  Q  L  K  P  G  T  S
181 CACGAACGCCTACACGGCGAACCTCTTCTTCAACCAGGCCCAGCTCAAGCCAGGGACTTC 240
    ----:----|----:----|----:----|----:----|----:----|----:----|
     E  K  L  T  A  I  V  P  G  I  S  P  E  D  L  R  A  M  Q  R
241 GGAGAAGCTGACTGCGATCGTGCCAGGCATCTCGCCAGAAGATCTCCGAGCCATGCAGCG 300
    ----:----|----:----|----:----|----:----|----:----|----:----|
     A  P  M  R  G  F  G  P  A  I  L  Q  E  P  N  V  Y  R  T  Y
301 CGCGCCGATGCGCGGCTTCGGCCCCGCCATCCTCCAGGAGCCCAACGTCTACCGGACGTA 360
    ----:----|----:----|----:----|----:----|----:----|----:----|
     E  A  V  T  H  Y  G  E  A  I  K  A  L  I  N  E  Q  C  G  D
361 CGAGGCCGTGACGCACTACGGGGAGGCCATCAAGGCGCTGATCAACGAGCAGTGCGGGGA 420
    ----:----|----:----|----:----|----:----|----:----|----:----|
     G  I  M  S  A  I  D  F  Y  L  D  V  G  T  T  T  G  K  K  G
421 CGGCATCATGAGCGCCATCGACTTCTATCTGGACGTAGGCACGACCACCGGCAAGAAGGG 480
    ----:----|----:----|----:----|----:----|----:----|----:----|
     E  K  R  V  V  I  T  M  N  G  K  F  L  P  H  I  E  Q  V  A
481 CGAGAAGCGCGTGGTCATCACCATGAACGGCAAGTTCCTGCCCCACATTGAGCAGGTGGC 540
    ----:----|----:----|----:----|----:----|----:----|----:----|
     A  D  N  T  V  P  G  P  R  D  *
541 GGCCGACAACACGGTGCCCGGCCCGAGGGACTGAGCCGCAAGCGGGTGGAGAGCGGCGCT 600

601 GCGGCCGCTGCAGGGCAGTTTGCTGCAGGACATCGCGTGGGTTCTGCACCCTCAAAAAAA 660

661 AAAAAAAA 668
```
