# Supplementary material for: Diversity, prevalence, and expression of cyanase genes (cynS) in planktonic marine microorganisms
Source: ISME J. 2021 Aug 18;16(2):602–5. doi: 10.1038/s41396-021-01081-y (PMC8776842; doi:10.1038/s41396-021-01081-y)
Supplement: Supplementary file 11 — Supplementary figure 2 [file 41396_2021_1081_MOESM11_ESM.pdf]

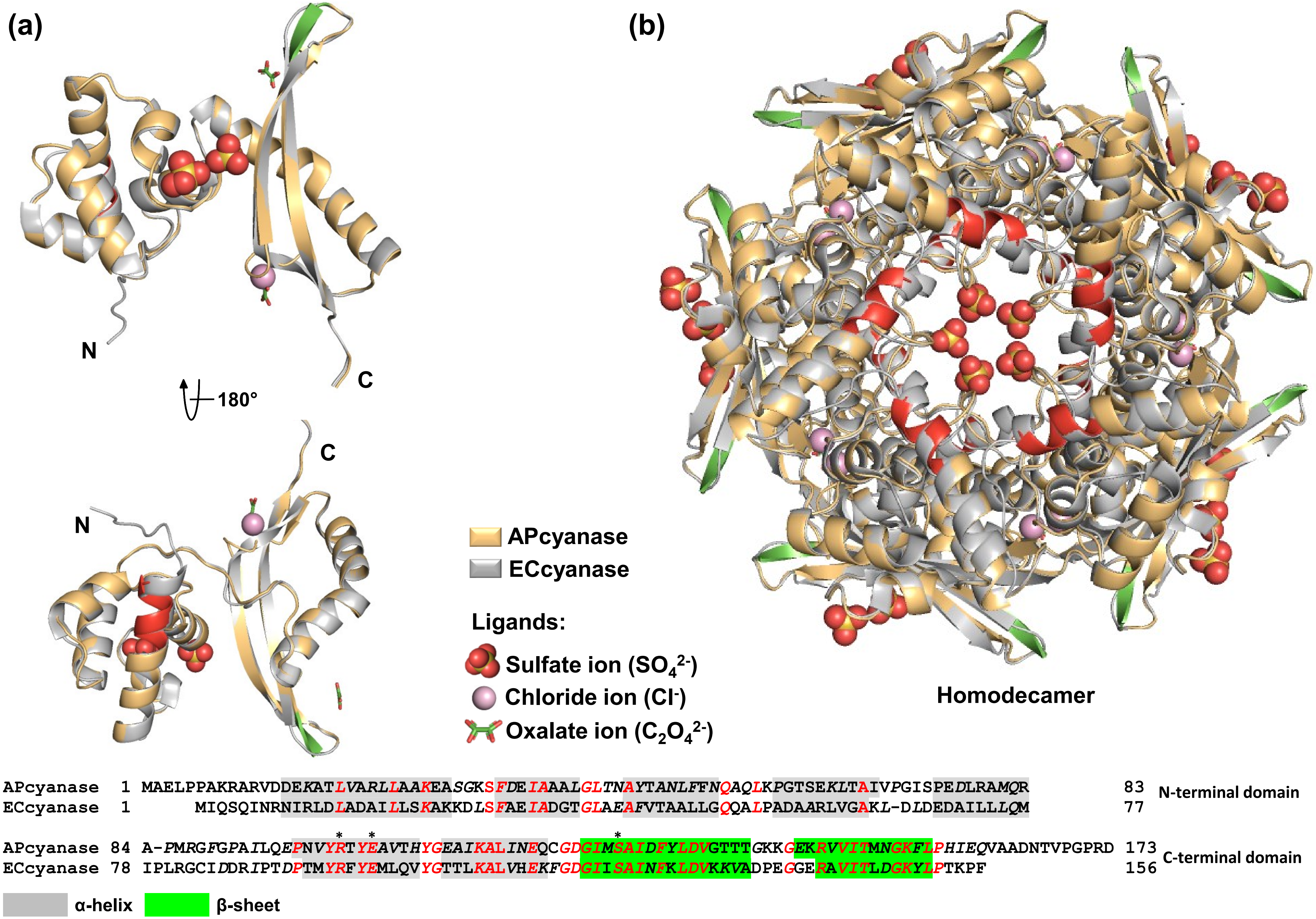

(a)
(b)
N
C
180°
C
N
APcyanase
ECcyanase
Ligands:
Sulfate ion (SO_4^{2-})
Chloride ion (Cl^-)
Oxalate ion ($C_2O_4^{2-}$)
Homodecamer
APcyanase 1 MAELPPAKRARVDDEKATLVARLLAAKEASGKSFDEIAAALGLTNAYTANLFFNQAQLKPGTSEKLTAIVPGISPEDLRAMQR 83
ECcyanase 1 MIQSQINRNIRLDLADAILLSKAKKDLSFAEIADGTGLAEAFVTAALLGQQALPADAARLVGAKL-DLDEDAILLLQM 77
N-terminal domain
* * *
APcyanase 84 A-PMRGFGPAILQEPNVYRTYEAVTHYGEAIKALINEQCGDGIMSAIDFYLDVGTTTGKKGEKRVVITMNGKFLPHIEQVAADNTVPGPRD 173
ECcyanase 78 IPLRGCIDDRIPTDPTMYRFYEMLQVYGTTLKALVHEKFGDGIISAINFKLDVKKVADPEGGERAVITLDGKYLPTKPF 156
C-terminal domain
α-helix
β-sheet
