# Supplementary figures and images for: Diversity, prevalence, and expression of cyanase genes (cynS) in planktonic marine microorganisms
Source: ISME J. 2021 Aug 18;16(2):602–5. doi: 10.1038/s41396-021-01081-y (PMC8776842; doi:10.1038/s41396-021-01081-y)

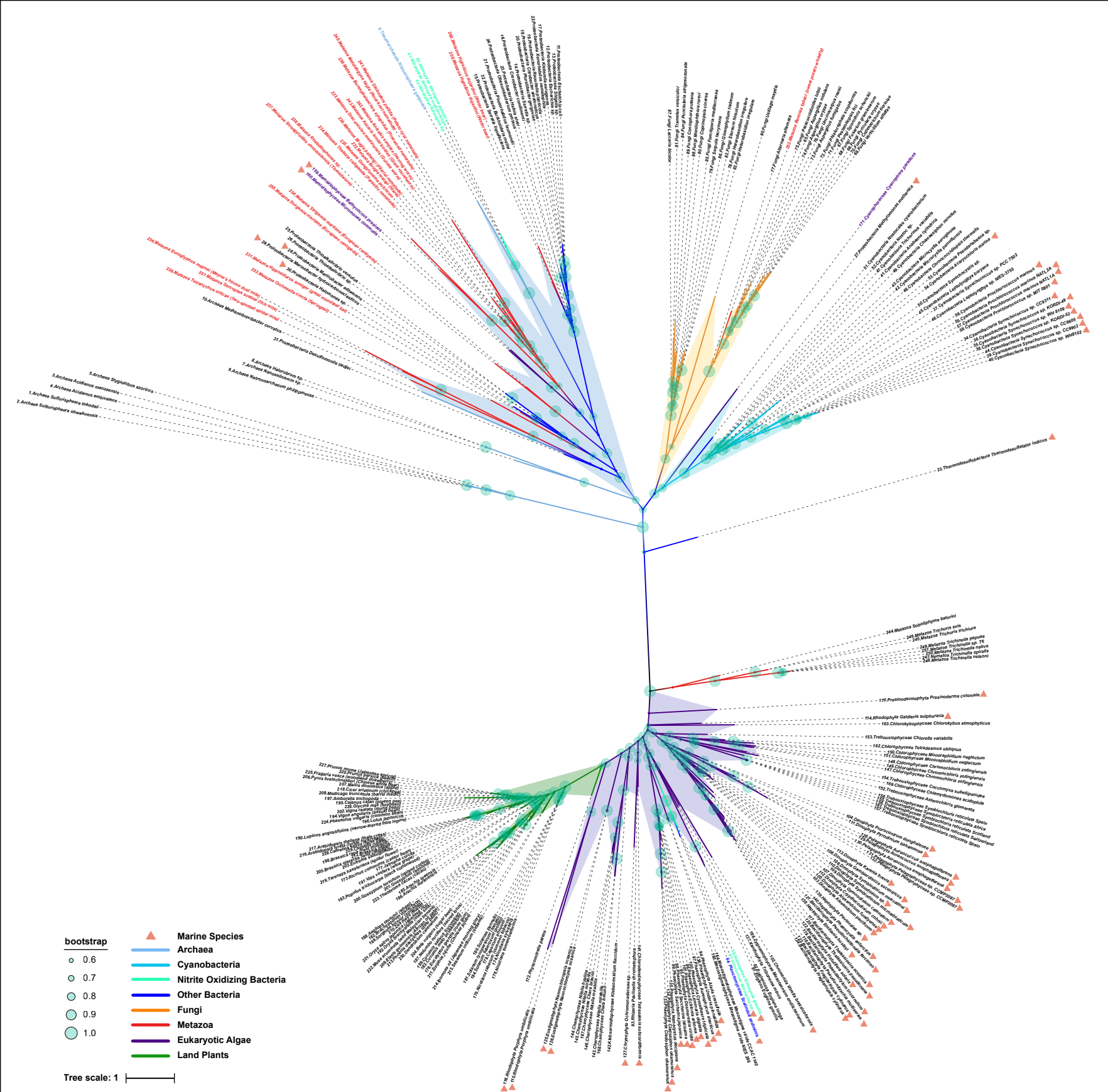

Supplement: Supplementary file 12 — Supplementary figure 3 [file 41396_2021_1081_MOESM12_ESM.pdf]

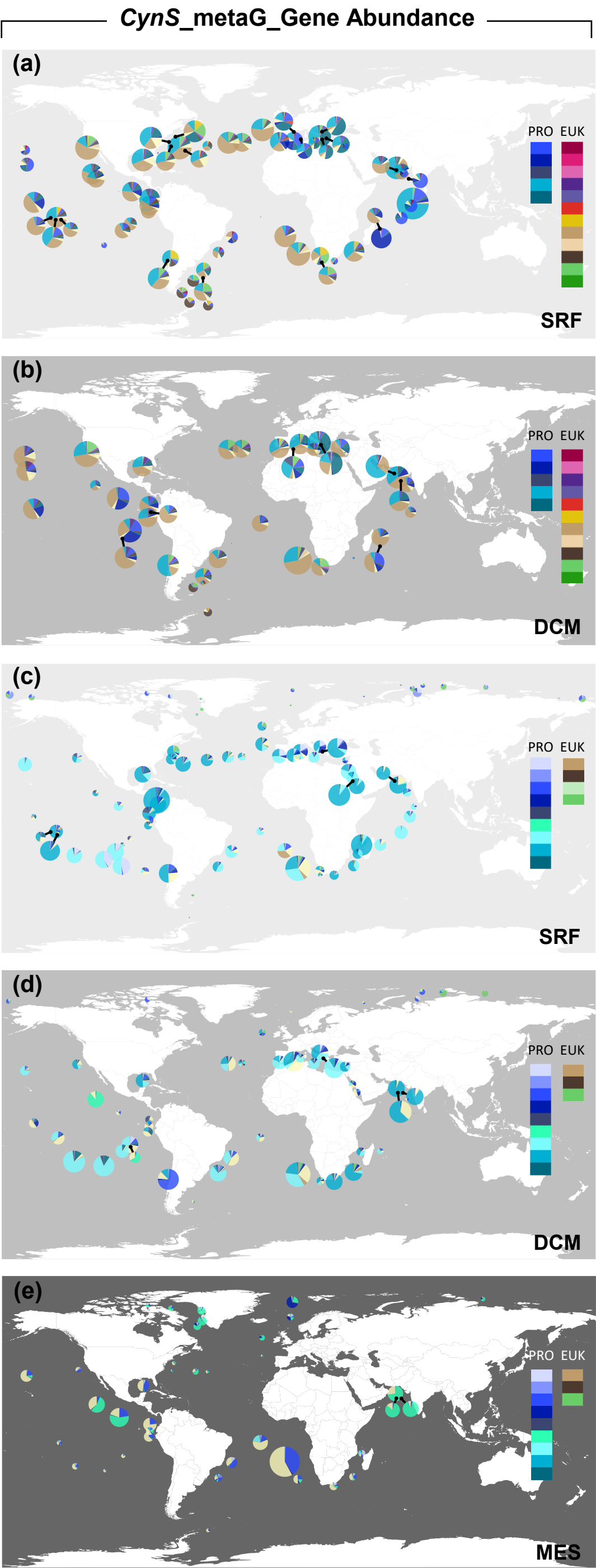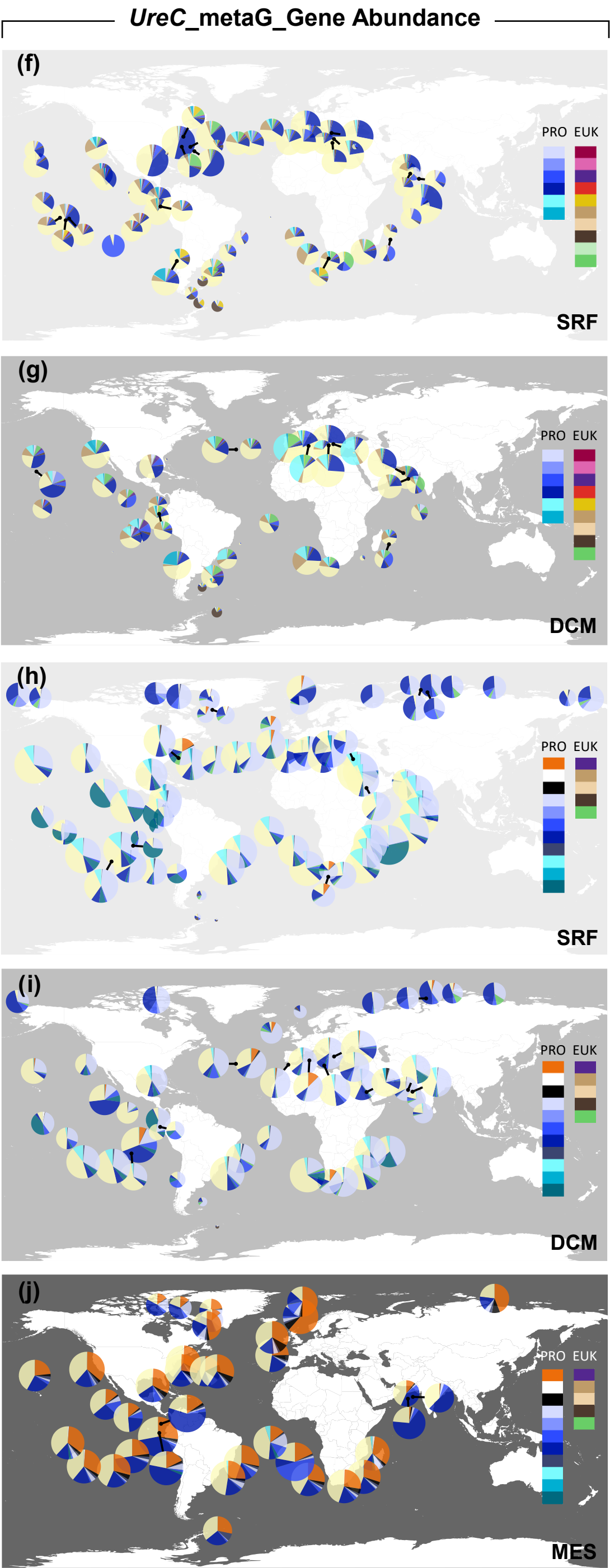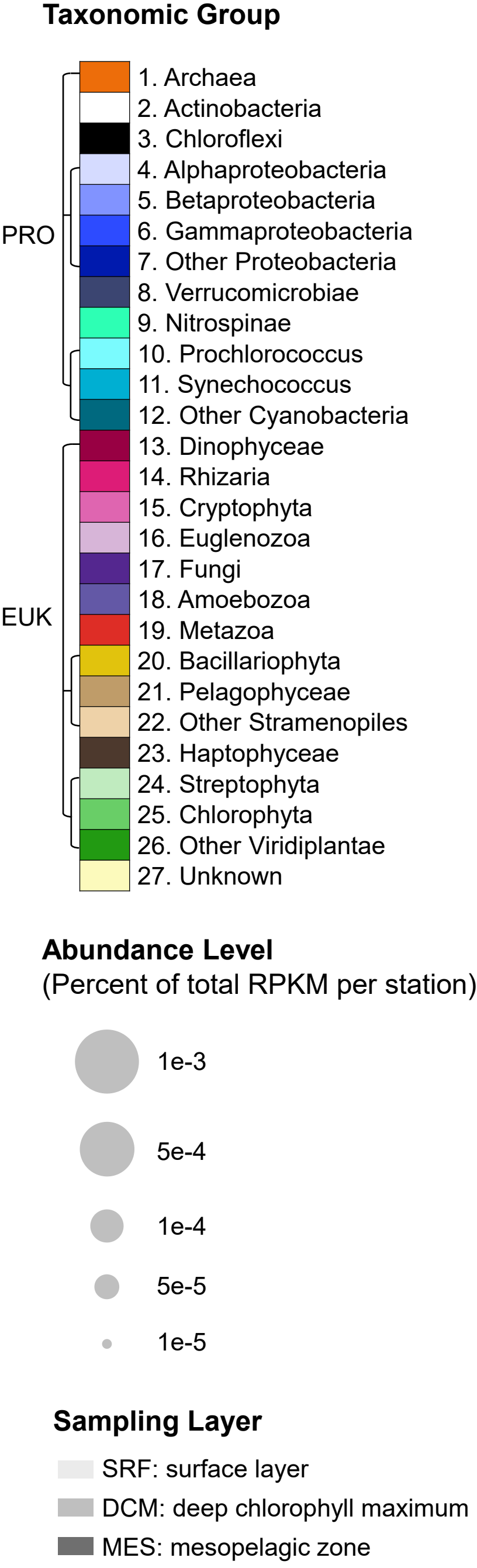

Supplement: Supplementary file 13 — Supplementary figure 4 [file 41396_2021_1081_MOESM13_ESM.pdf]
