# Supplementary material for: Diversity, prevalence, and expression of cyanase genes (cynS) in planktonic marine microorganisms
Source: ISME J. 2021 Aug 18;16(2):602–5. doi: 10.1038/s41396-021-01081-y (PMC8776842; doi:10.1038/s41396-021-01081-y)
Supplement: Supplementary file 14 — Supplementary figure 5 [file 41396_2021_1081_MOESM14_ESM.pdf]

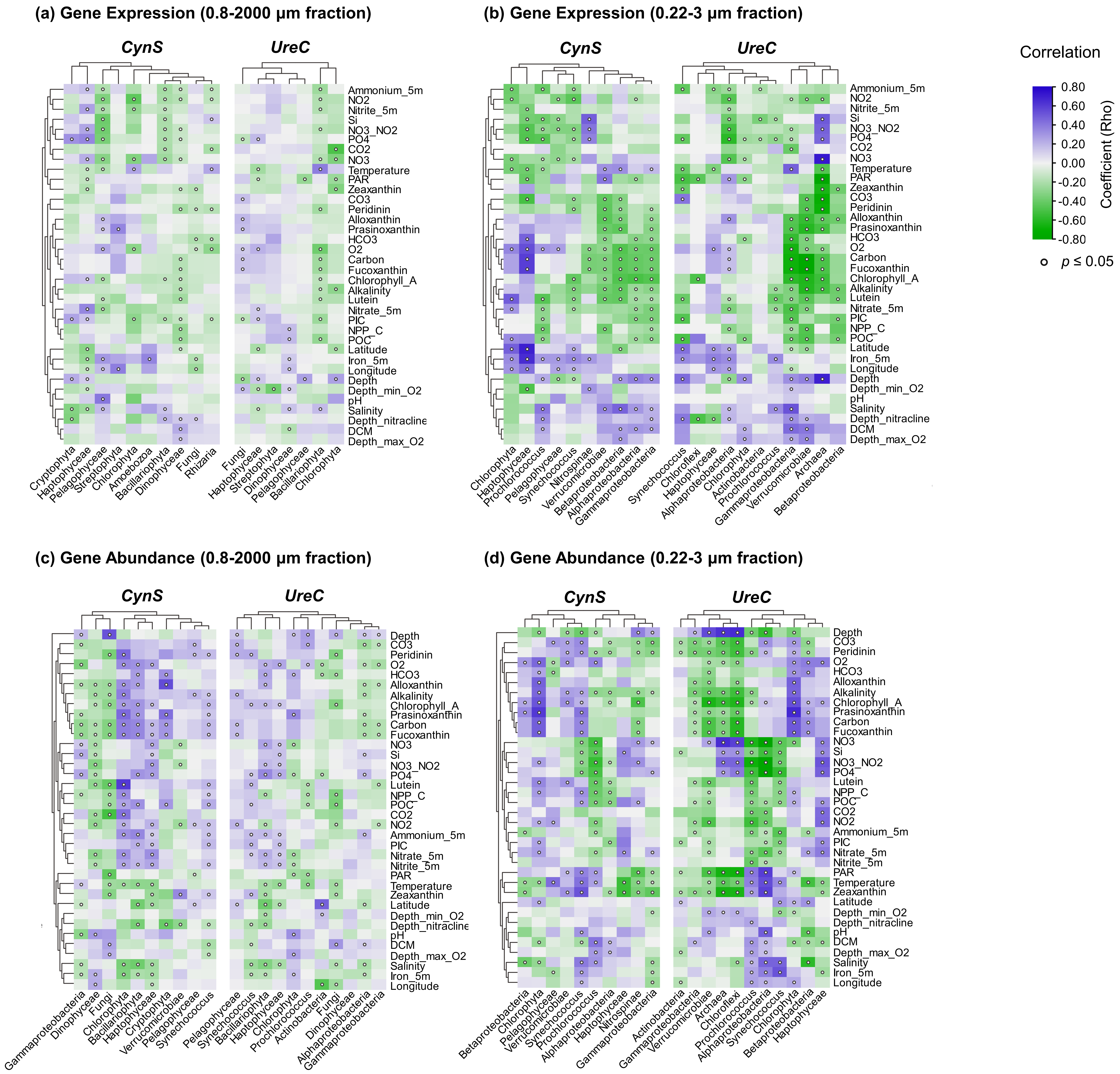
(a) Gene Expression (0.8-2000 µm fraction)
CynS
UreC
Ammonium_5m
NO2
Nitrite_5m
Si
NO3_NO2
PO4
CO2
NO3
Temperature
PAR
Zeaxanthin
CO3
Peridinin
Alloxanthin
Prasinoxanthin
HCO3
O2
Carbon
Fucoxanthin
Chlorophyll_A
Alkalinity
Lutein
Nitrate_5m
PIC
NPP_C
POC
Latitude
Iron_5m
Longitude
Depth
Depth_min_O2
pH
Salinity
Depth_nitracline
DCM
Depth_max_O2
Cryptophyta
Haptophyceae
Pelagophyceae
Streptophyta
Chlorophyta
Amoebozoa
Bacillariophyta
Dinophyceae
Fungi
Rhizaria
Fungi
Haptophyceae
Streptophyta
Dinophyceae
Pelagophyceae
Bacillariophyta
Chlorophyta
(b) Gene Expression (0.22-3 µm fraction)
CynS
UreC
Ammonium_5m
NO2
Nitrite_5m
Si
NO3_NO2
PO4
CO2
NO3
Temperature
PAR
Zeaxanthin
CO3
Peridinin
Alloxanthin
Prasinoxanthin
HCO3
O2
Carbon
Fucoxanthin
Chlorophyll_A
Alkalinity
Lutein
Nitrate_5m
PIC
NPP_C
POC
Latitude
Iron_5m
Longitude
Depth
Depth_min_O2
pH
Salinity
Depth_nitracline
DCM
Depth_max_O2
Chlorophyta
Haptophyceae
Prochlorococcus
Pelagophyceae
Synechococcus
Nitrospinae
Verrucomicrobiae
Betaproteobacteria
Alphaproteobacteria
Gammaproteobacteria
Synechococcus
Chloroflexi
Haptophyceae
Alphaproteobacteria
Chlorophyta
Actinobacteria
Prochlorococcus
Gammaproteobacteria
Verrucomicrobiae
Archaea
Betaproteobacteria
Correlation
0.80
0.60
0.40
0.20
0.00
-0.20
-0.40
-0.60
-0.80
Coefficient (Rho)
p ≤ 0.05
(c) Gene Abundance (0.8-2000 µm fraction)
CynS
UreC
Depth
CO3
Peridinin
O2
HCO3
Alloxanthin
Alkalinity
Chlorophyll_A
Prasinoxanthin
Carbon
Fucoxanthin
NO3
Si
NO3_NO2
PO4
Lutein
NPP_C
POC
CO2
NO2
Ammonium_5m
PIC
Nitrate_5m
Nitrite_5m
PAR
Temperature
Zeaxanthin
Latitude
Depth_min_O2
Depth_nitracline
pH
DCM
Depth_max_O2
Salinity
Iron_5m
Longitude
Gammaproteobacteria
Dinophyceae
Fungi
Chlorophyta
Bacillariophyta
Haptophyceae
Cryptophyta
Verrucomicrobiae
Pelagophyceae
Synechococcus
Pelagophyceae
Synechococcus
Bacillariophyta
Haptophyceae
Chlorophyta
Prochlorococcus
Actinobacteria
Fungi
Dinophyceae
Alphaproteobacteria
Gammaproteobacteria
(d) Gene Abundance (0.22-3 µm fraction)
CynS
UreC
Depth
CO3
Peridinin
O2
HCO3
Alloxanthin
Alkalinity
Chlorophyll_A
Prasinoxanthin
Carbon
Fucoxanthin
NO3
Si
NO3_NO2
PO4
Lutein
NPP_C
POC
CO2
NO2
Ammonium_5m
PIC
Nitrate_5m
Nitrite_5m
PAR
Temperature
Zeaxanthin
Latitude
Depth_min_O2
Depth_nitracline
pH
DCM
Depth_max_O2
Salinity
Iron_5m
Longitude
Betaproteobacteria
Chlorophyta
Pelagophyceae
Verrucomicrobiae
Synechococcus
Prochlorococcus
Alphaproteobacteria
Haptophyceae
Nitrospinae
Gammaproteobacteria
Actinobacteria
Gammaproteobacteria
Verrucomicrobiae
Archaea
Chloroflexi
Prochlorococcus
Alphaproteobacteria
Synechococcus
Chlorophyta
Betaproteobacteria
Haptophyceae
